# A clinical solution for non‐toxic 3D‐printed photon blocks in external beam radiation therapy

**DOI:** 10.1002/acm2.14225

**Published:** 2024-01-11

**Authors:** Joseph B. Schulz, Piotr Dubrowski, Clinton Gibson, Amy S. Yu, Lawrie Basil Skinner

**Affiliations:** ^1^ Department of Radiation Oncology School of Medicine Stanford University Stanford California USA

**Keywords:** 3D‐printed beam shaping, 3D‐printing, patient specific, photon blocks, tungsten BBs

## Abstract

**Purpose:**

A well‐known limitation of multi‐leaf collimators is that they cannot easily form island blocks. This can be important in mantle region therapy. Cerrobend photon blocks, currently used for supplementary shielding, are labor‐intensive and error‐prone. To address this, an innovative, non‐toxic, automatically manufactured photon block using 3D‐printing technology is proposed, offering a patient‐specific and accurate alternative.

**Methods and materials:**

The study investigates the development of patient‐specific photon shielding blocks using 3D‐printing for three different patient cases. A 3D‐printed photon block shell filled with tungsten ball bearings (BBs) was designed to have similar dosimetric properties to Cerrobend standards. The generation of the blocks was automated using the Eclipse Scripting API and Python. Quality assurance was performed by comparing the expected and actual weight of the tungsten BBs used for shielding. Dosimetric and field geometry comparisons were conducted between 3D‐printed and Cerrobend blocks, utilizing ionization chambers, imaging, and field geometry analysis.

**Results:**

The quality assurance assessment revealed a −1.3% average difference in the mass of tungsten ball bearings for different patients. Relative dose output measurements for three patient‐specific blocks in the blocked region agreed within 2% of each other. Against the Treatment Planning System (TPS), both 3D‐printed and Cerrobend blocks agreed within 2%. For each patient, 6 MV image profiles taken through the 3D‐printed and Cerrobend blocks agreed within 1% outside high gradient regions. Jaccard distance analysis of the MV images against the TPS planned images, found Cerrobend blocks to have 15.7% dissimilarity to the TPS, while that of the 3D‐printed blocks was 6.7%.

**Conclusions:**

This study validates a novel, efficient 3D‐printing method for photon block creation in clinical settings. Despite potential limitations, the benefits include reduced manual labor, automated processes, and greater precision. It holds potential for widespread adoption in radiation therapy, furthering non‐toxic radiation shielding.

## 1 INTRODUCTION

Remarkable strides have been made in improving the physical attributes of external beam radiation therapy treatment delivery in recent years.^1^ A critical driver of this progress lies in the burgeoning realm of additive manufacturing, or more generally 3D‐printing, and its consequential applications in radiation oncology.^2^, ^3^ This technology ushers in an era of efficient and cost‐effective patient‐specific solutions, encompassing custom bolus, phantoms, and electron cutouts.^4^, ^5^, ^6^, ^7^, ^8^

In radiation oncology, clinical plans frequently utilize a multi‐leaf collimator (MLC) to modulate the radiation beam, thereby maximizing normal tissue sparing.^9^ Nevertheless, clinical necessities can sometimes surpass the physical capacity of the MLC, owing to intrinsic inter‐leaf leakage, intra‐leaf leakage, and geometric constraints attributed to the unique shaping of the leaves. A prime example is mantle region therapy, where the MLCs fall short in fully safeguarding normal tissues within a single field.

Currently, the solution is for supplementary shielding to be manufactured and implemented in the treatment, predominantly in the form of Cerrobend photon blocks. However, this remedy is not without its share of obstacles. The process of fabricating a Cerrobend photon block is laborious and fraught with challenges, including the likelihood for the Cerrobend to crack if poured all at once. A workaround to this problem entails pouring the Cerrobend into the mold in successive layers, each succeeding layer poured after the preceding one has cooled. However, this method introduces the risk of air bubble formation between layers. Additionally, often the blocks are made larger than their in‐field shape to facilitate the technician attaching two bolts per Cerrobend part to the mounting tray, with the bolts being placed at least 3 cm apart. The entire manufacturing process, due to its complexity and labor intensity, presents numerous opportunities to compromise dosimetric accuracy, including issues with the design's divergence and overall resolution.

Consequently, there is a compelling need to devise an innovative, non‐toxic, and automatically manufactured photon block. We aim to extend the use of 3D‐printing in radiation oncology by offering a comprehensive overview of automatically generated tungsten ball bearing (BB) filled 3D‐printed photon block shell that is not only patient‐specific but also non‐toxic.

## 2 MATERIALS AND METHODS

### 2.1 Patient plan selection

**FIGURE 1:**
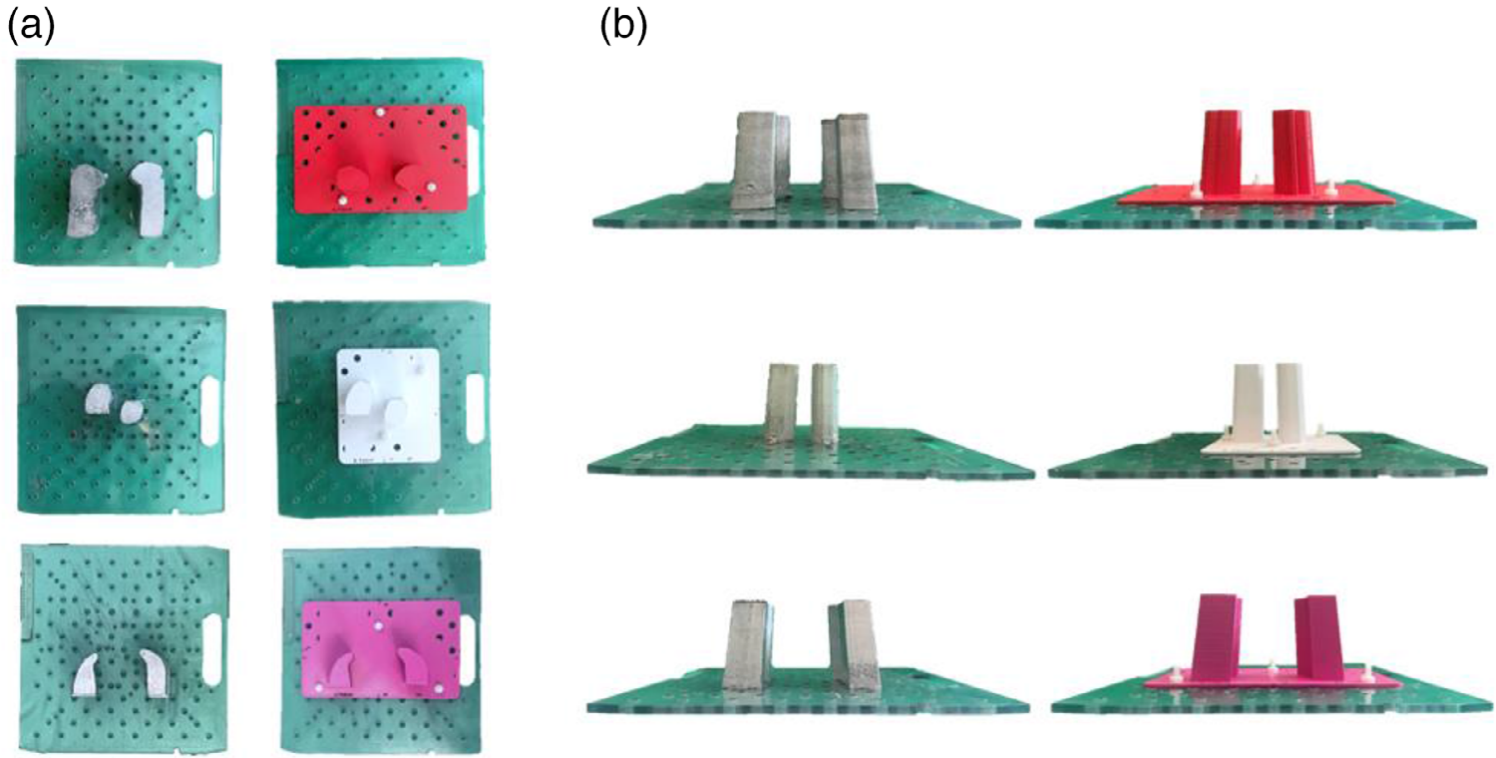
(a) Top‐down view of three patient photon blocks. The rows from top to bottom correspond to patients A, B, and C, respectively, while left column is Cerrobend and right is 3D printed. Note the Cerrobend blocks A, C are extended inferiorly out of the field to allow for additional mounting screws. (b) Side view of the three patient photon blocks in the same row, column arrangement as (a).

Photon blocks are created by dosimetrists in the treatment planning system (TPS) Varian Eclipse v15.6 (Varian Medical Systems, Siemens Healthineers, Palo Alto, CA) by creating a margin around the specific anatomy to be blocked in a particular field. Three patient‐specific photon shielding blocks were chosen for investigation: nearly circular mantle field lung shielding (patient A), pelvis field ovary shielding (patient B), and long, thin mantle field lung shielding (patient C) (Figure 1, Table 1). The dosimetric parameters of point output between the blocks, point output under one of the blocks, megavoltage (MV) image profiles, and field geometry were investigated for clinically used Cerrobend blocks and 3D‐printed blocks.

**TABLE 1:** Patient‐specific cases details.

Patient	Treatment site	Shielded site	Field orientation
A	Mantle	Lungs	Anterior‐Posterior
B	Pelvis	Ovaries	Anterior‐Posterior
C	Mantle	Lungs	Posterior‐Anterior

### 2.2 3D‐printed photon block design

**FIGURE 2:**
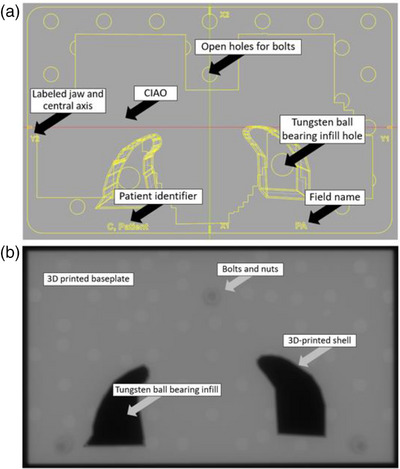
(a) A top‐down wire‐frame view of the automatically generated computer‐aided‐design model of the 3D‐printed photon block, with features noted. (b) A 6 megavolt image of the 3D‐printed photon block, with features noted. The 3D‐printed baseplate is 1 cm larger than the treatment field in all jaw directions.

The complete 3D‐printed photon block shell design features five general components: the baseplate, the tungsten BB containing shell, checkerboard hole pattern, the blocked complete irradiated area outline (CIAO), and aluminum nuts and bolts. The aluminum bolts are placed outside the CIAO. Although aluminum was chosen, since they are outside the CIAO, any metal may be used for the bolts. Plastic bolts are not recommended due to low shear strength. These 3D‐printed and reusable components in conjunction with the tungsten BB infill compose the overall 3D‐printed photon block. The 3D‐printed photon block is designed to have similar dosimetric properties to the currently used Cerrobend standard. We are limited in design due to the weight and the amount of material needed to equivalently attenuate the beam. We are also constrained by the dimensions allowed between a Varian TrueBeam applicator mount tray insert and the head of the gantry which is approximately 8.2 cm. The 3D‐printed photon block is attached to the provided Varian applicator tray by inserting the aluminum nuts and bolts through the baseplate and the tray. The 3D‐printed block shell used for containing the tungsten BBs were designed to be automatically calculated to the appropriate divergence for the beam source (Figure 2). The tungsten BB containing block shell has an interior thickness of 8 cm, whereas the Cerrobend blocks are made at 7.5 cm thickness. After initial testing with thicker walls, a final block shell wall thickness of 1.2 mm was chosen. The choice of wall thickness is a compromise between strength and dosimetric impact (see Sections 3.1, 4.2 for structural integrity tests). Thickness of the tungsten ball bearing layer was adjusted to match the treatment planning system. The shell walls were not incorporated in the treatment planning system's dose calculation. The baseplate was designed to extend 1 cm larger in all jaw directions at a 100 cm source‐to‐surface distance (SSD). The tungsten BB‐filled block shells were sealed by affixing a reusable 3D‐printed cylindrical plug into the fill hole, which was then secured in place between the 3D‐printed baseplate and the mounting tray. The final design of the automatically generated 3D‐printed photon block shell emerged after an iterative review and redesign process involving physicists, dosimetrists, radiation therapists, and physicians, ensuring consideration and clinical viability.

### 2.3 3D‐printed photon block generation

The scripted generation of the patient‐specific photon shielding block shells for 3D‐printing is completed via the Eclipse Scripting API (ESAPI) as well as the Python programming language, and directly accessible within the clinical TPS. Patient‐specific attributes, such as the spatial coordinates of the block's perimeters are interfaced (Table 2). A stereolithography (STL) file is exported to a patient‐specific file directory for 3D‐printing. The automatic generation is completed in approximately 20 s.

**TABLE 2:** Data interfaced from the ESAPI to Python.

Parameter	ESAPI source
Patient identifier	Patient.LastName, Patient.FirstName
Field name	ExternalPlanSetup.Beams.Id
Blocks	ExternalPlanSetup.Beams.Blocks.Outline
CIAO	ExternalPlanSetup.Beams.ControlPoints.LeafPositions
X1 Jaw	ExternalPlanSetup.Beams.ControlPoints.JawPositions.X1
X2 Jaw	ExternalPlanSetup.Beams.ControlPoints.JawPositions.X2
Y1 Jaw	ExternalPlanSetup.Beams.ControlPoints.JawPositions.Y1
Y2 Jaw	ExternalPlanSetup.Beams.ControlPoints.JawPositions.Y2

### 2.4 3D‐printed photon block fabrication

The 3D‐printed photon block shell STL files are imported into Bambu Studio v1.7, an open source 3D‐printing slicing software, and remotely set to and printed with tough PLA (MatterHackers PRO Series PLA, MatterHackers, Lake Forest, CA, USA) via a fused deposition modeling (FDM) type Bambu Lab X1 Carbon (Bambu Lab, Austin, TX, USA). The FDM 3D‐printer is configured with a 0.6 mm nozzle, ensuring at least two walls are deposited for each of the BB containing shells of 1.2 mm thickness. The infill was set to 30%, with three top and bottom walls, which only effects the baseplate and top face of the 3D‐printed photon block shell. The baseplate was oriented flat onto the print bed. The print time for each patient‐specific photon shielding device was approximately 1.5 h.

### 2.5 3D‐printed photon block quality assurance

The shell was filled compactly with 1.5–2 mm tungsten BBs for radiation shielding. This has been shown to be adequate for clinical radiation shielding.^7^, ^8^ The expected weight of the tungsten BBs was calculated and compared to the actual value. The shape of the tungsten BB containing shell is assumed as a frustum for the purpose of calculating the mass of the tightly packed tungsten BBs. First, the area, *A*, of a block's bottom face, $A_{\mathrm{bottom}}$ and the top face, $A_{top}$, are calculated for each of the two blocks. The area of the bases are calculated through the shoelace theorem with the input being the n clockwise‐ordered spatial coordinates, $x_{i}$, $y_{i}$, of the perimeter of the blocks at 100 cm SSD, directly interfaced via ESAPI (Equation 1).

(1) $$A\left(x_{i},y_{i},x_{i+1},y_{i+1},n\right)=\frac{1}{2}\;\sum_{i}\left|\begin{array}{cc} x_{i} & y_{i}\\ x_{i+1} & y_{i+1} \end{array}\right|$$

Secondly, the volume of each block is calculated, where h is the height of the frustum, and the previously calculated areas of the top, $A_{top}$, and bottom face, $A_{bottom}$, are inputted per block (Equation 2).

(2) $$\begin{array}{c} V\left(h,A_{top},A_{bottom}\right)=\frac{h}{3}\left(A_{top}+\sqrt{A_{top}A_{bottom}}+A_{bottom}\right) \end{array}$$

The total mass, *M*, of *n* total theoretical tungsten BB containing shells are then calculated, from the sphere packing fraction η (approximately 0.6 from theory of randomly packed spheres^10^, ^11^ and our prior work^7^), the density of the tungsten alloy BBs ρ (approximately 17.6 g/cc) and $V_{i}$ which represents the volume of each $i^{th}$ BB containing shell utilizing the previously validated parameters (Equation 3).^7^

(3) $$M=\eta \cdot \rho \cdot \sum_{i}V_{i}$$

The constants η and ρ combine to give average density of the BB‐filled volume. This is approximately 10.5 g/cc, from random sphere packing theory, but to obtain a more precise predicted mass for a given 3D‐printing process and batch of BB's one should obtain a calibrated value for the product ηρ by weighing a set of fully‐filled representative blocks. The calculated mass is then compared to the mass difference of the fully filled tungsten BB 3D‐printed photon block shell and the 3D‐prrinted photon block shell with the absence of BBs (only the 3D‐printed block shell). The BB‐filled volumes ($V_{i}$) were calculated at the Python stage of block shell generation. The Python script converts the design to STL format in the last step. It is possible, though less convenient, to also calculate $V_{i}$ through Boolean and trimming operations of final STL shell volumes.

### 2.6 Point dose measurement

Point dose output measurements were taken in solid water using a pinpoint ionization chamber (PTW PinPoint chamber, PTW‐Frieburg, Breisgau, Germany) with a Varian TrueBeam. The setup for the linac energies of 6, 10, and 15 MV were taken at 100 cm SSD, 5 cm of solid water depth and 5 cm of solid water backscatter. For all measurements, 400 monitor units (MUs) were delivered. For each patient's blocks, a baseline measurement was taken at the central axis (CAX) with the clinical jaw size. The ion chamber was then laterally and longitudinally shifted between each patient's photon blocks, the clinical field is then configured with MLC and jaw, and measurements were taken for the Cerrobend then 3D‐printed photon blocks. The ion chamber was then laterally and longitudinal shifted under one of the photon blocks for each patient with their clinical field configured, for the Cerrobend then 3D‐printed photon blocks.

The dose for each photon energy was calculated in corresponding setup conditions at the CAX, the patient‐specific between block location, and patient‐specific under block location utilizing the Eclipse TPS and Acuros v15.6. The ratio of the CAX measurement to the between block location measurement is computed and compared to the 3D‐printed and Cerrobend ratio, with the overall difference to TPS computed. The ratio of the CAX measurement to the under block measurement is also computed and compared to the 3D‐printed and Cerrobend ratio, where both are then compared to the TPS.

### 2.7 MV image analysis

The Electronic Portal Imaging Device (EPID) imager on‐board the TrueBeam was used to capture MV images of both the 3D‐printed and Cerrobend blocks for each patient. For each image, the clinical field was used, including gantry orientation, and 100 MUs were delivered. The MV images for the 3D‐printed and Cerrobend design are overlayed and profiles are taken through each of the blocks for each patient. Images at all four cardinal angles were taken to further evaluate any sag or shift in the blocks.

### 2.8 Photon block field geometry

A field geometry comparison between the 3D‐printed and Cerrobend photon blocks was performed on 6 MV images taken at 100 cm SSD. The resulting Digital Imaging and Communications in Medicine (DICOM) images are thresholded and binarized using MATLAB R2023b (MathWorks, Natick, MA, USA) to maintain spatial accuracy. The TPS block perimeters used for the generation and fabrication of each patient's 3D‐printed photon block are also masked and binarized. The patient field CIAO from the TPS was also imported with ESAPI, then masked over both MV images and the TPS blocks to account for Cerrobend blocks enlarged size outside of the treatment field. Utilizing the built‐in “jaccard” function, the Jaccard distance is applied to measure the dissimilarity of sample sets and is described in Equation (4), where A and B in this context are the set of binarized pixels of the blocked area inside the CIAO, which is defined by the MLC. This then excludes any extended area of the Cerrobend blocks outside the CIAO field, which may be added to allow for screwing to the baseplate. The set of pixels A is either the attributed to the 3D‐printed design (3D) or the Cerrobend design (CB), and B is the TPS design; delineated respectively, as $d_{j}\left(3D,TPS\right)\;$and $d_{j}\left(CB,TPS\right)$, where $0\leq d_{j}\left(A,B\right)\leq 1$.

(4) $$d_{j}\left(A,B\right)=\frac{\left|\left(A\cup B\right)\right|-\left|\left(A\cap B\right)\right|}{\left|\left(A\cup B\right)\right|}$$

## 3 RESULTS

### 3.1 Quality assurance

The calculated mass, measured mass, and the percentage difference of the tungsten BBs are shown in Table 3. The average percentage difference was −1.26%. Patient C exhibited the highest individual block shell BB volume at 92 cc, whereas patient B had the smallest volume at 50 cc. MV images captured at 0 and 180 degrees were compared through image registration and showed no discernible shift. Likewise, image registration between gantry angle 0 and 90 or 270 revealed between 0 and 0.3 mm sag or shift (see discussion in Section 4.2).

**TABLE 3:** Photon block tungsten ball bearing weight quality assurance.

Patient	Calculated mass [g]	Measured mass [g]	Percent difference [%]
A	1343.5 g	1361.9 g	−1.37%
B	1311.2 g	1329.6 g	−1.40%
C	1681.1 g	1698.2 g	−1.01%

### 3.2 Point dose measurement

**FIGURE 3:**
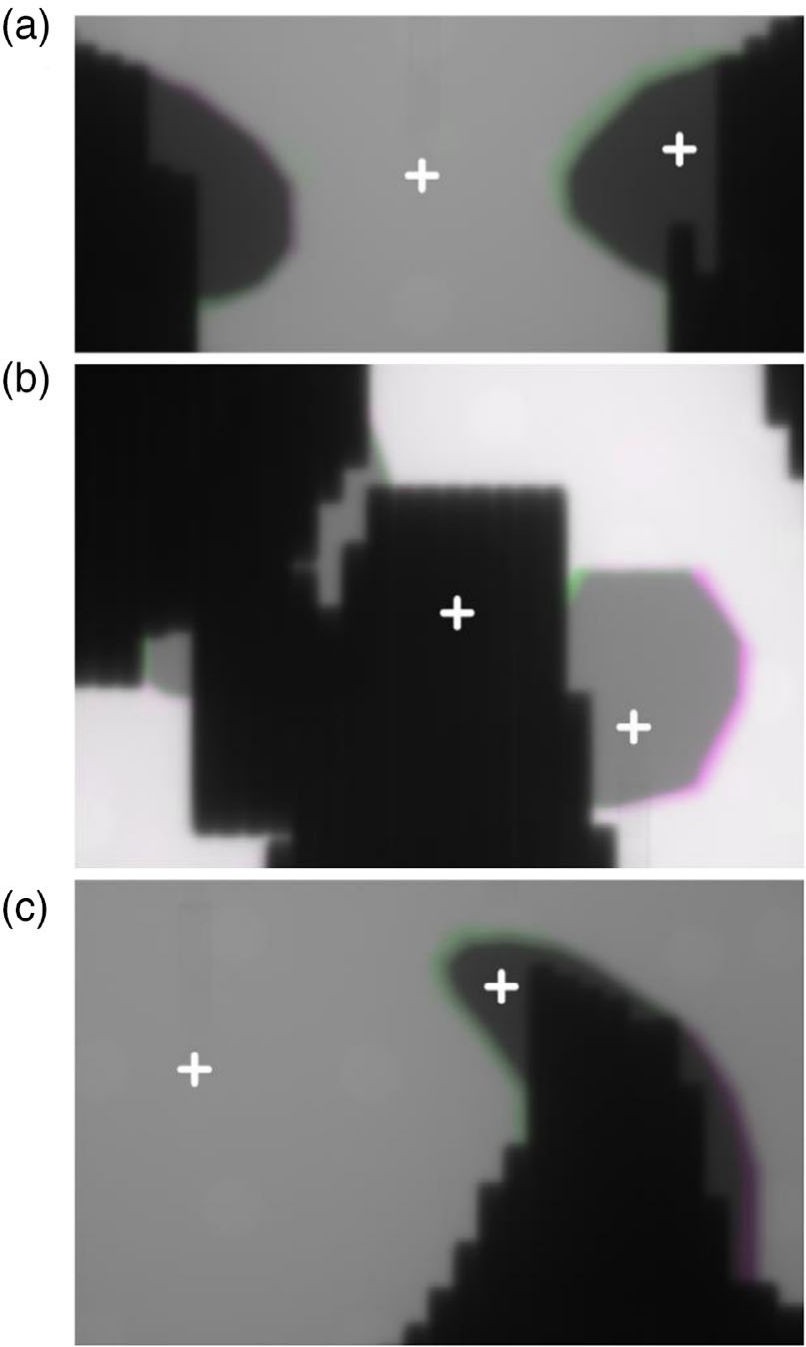
Megavoltage (MV) images taken during measurement with a pinpoint ion chamber either underneath the photon blocks, or between the photon blocks at clinically delivered MV energies of 6, 15, and 6 MV for patient A, B, and C, respectively. Where either the Cerrobend or 3D‐printed block extends outside of their intersection are represented as, respectively, as green and pink.

**FIGURE 4:**
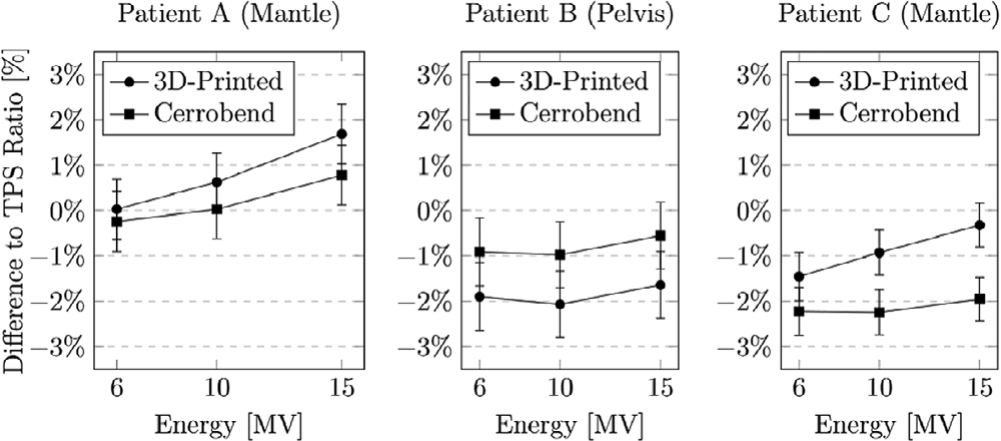
Percentage difference to the Treatment Planning System (TPS) ratio for three distinct patients using both 3D‐printed and Cerrobend blocks. Measurements were conducted under a photon block at linac energies of 6, 10, and 15 MV, using a PTW PinPoint ionization chamber within a 100 cm SSD, 5 cm solid water depth, and 5 cm solid water backscatter configuration.

**FIGURE 5:**
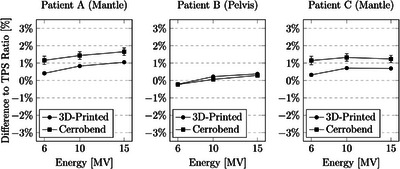
The percentage difference to the treatment planning system (TPS) ratio for three patients, using 3D‐printed and Cerrobend blocks. Measurements were conducted between the photon blocks at linac energies of 6, 10, and 15 MV, using a PTW PinPoint ionization chamber within a 100 cm SSD, 5 cm solid water depth, and 5 cm solid water backscatter configuration. For patient B, the measurements between the blocks were shielded by the multi‐leaf‐collimator.

The placement of each of the measurements are given in Figure 3. The relative dose output measurements for the three patient‐specific blocks were compared (Figures 4 and 5). When positioned under one of the photon blocks, the output of the blocks agreed within 2% of each other. When compared to the TPS, both the 3D‐printed and Cerrobend blocks were found to agree approximately within 2%. When position between the photon blocks, the output agreed within 2% when comparing both the 3D‐printed and Cerrobend to the TPS.

### 3.3 MV image analysis

**FIGURE 6:**
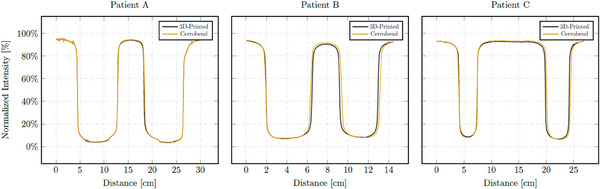
Overlayed megavoltage image line profiles through 3D‐printed and Cerrobend blocks for patient A, B, and C.

For each patient, 6 MV image profiles were overlayed and measured (Figure 6). For all patients, the profiles taken through the 3D‐printed and Cerrobend blocks agreed within 1% outside of high gradient regions.

### 3.4 Photon block field geometry

**FIGURE 7:**
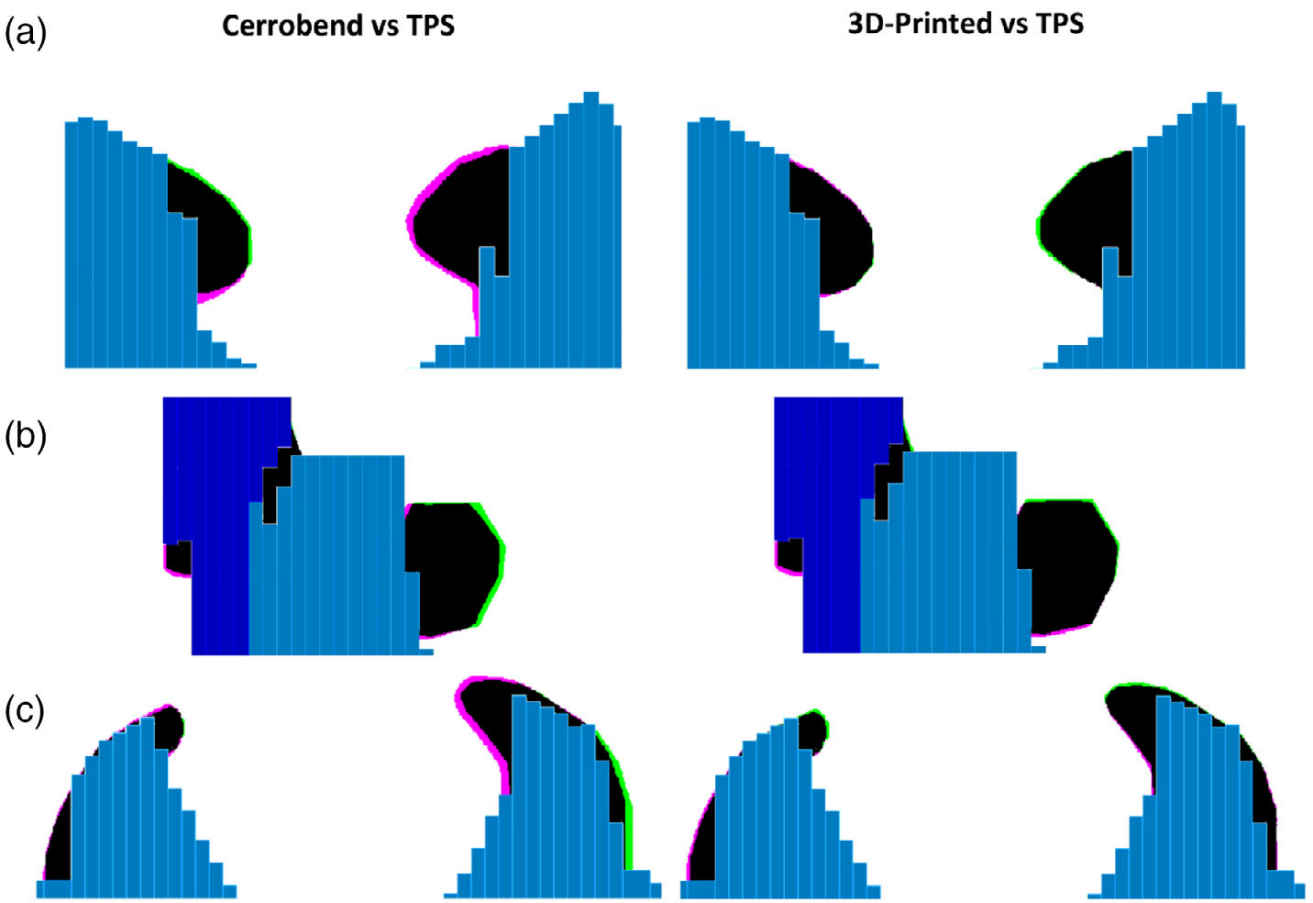
In the first column, overlayed in‐field block (black) of the Cerrobend (pink) and treatment planning system (TPS, green) block at 100 cm SSD. In the second column, overlayed in‐field block (black) of the 3D‐printed (pink) and treatment planning system (green) block at 100 cm SSD. The multi‐leaf collimator leaves (light blue, Bank A, dark blue, Bank B) from the TPS are masked over both Cerrobend and 3D‐printed blocks. Patient A, B, and C correspond to row (a), (b), and (c) respectively.

An in‐field block comparison at 100 cm SSD between the 3D‐printed, Cerrobend, and TPS aperture for each patient is shown in Figure 7. The computed values of the Jaccard distance are described in Table 4. On average, the 3D‐printed blocks had a Jaccard distance of 0.067, and the Cerrobend blocks had an average Jaccard distance of 0.157. Patient C's Cerrobend in‐field block deviated the most from the TPS.

**TABLE 4:** Jaccard distances for each patient case.

Patient	$d_{j}\left(CB,TPS\right)$	$d_{j}\left(3D,\mathrm{TPS}\right)$	$\frac{d_{j}\left(CB,TPS\right)}{d_{j}\left(3D,TPS\right)}\left[\%\right]$
A	0.143	0.036	397%
B	0.090	0.058	155%
C	0.239	0.108	221%

## 4 DISCUSSION

### 4.1 Dosimetric commissioning

The quality assurance procedure for packing the tungsten BBs in the 3D‐printed block shell has been demonstrated to be accurate within 1.5%. The calculation method for the mass, as described in Equation (3), could be fine‐tuned as more data points are gathered. If the BB‐filled block shell is overfilled, that is, the measured mass is higher than expected mass, this indicates either a small variance from the calibrated density of the BB filled volume (ηρ), or deviation of the shell volume from the expected volume. Since slightly higher attenuation is preferable to under‐attenuation, a looser tolerance on overweight may be applied compared to underweight, as long as the weight check QA can still catch gross “wrong shell volume” errors. The precise tolerance value is a clinical choice that should be made at commissioning.

The point dose measurements under the photon blocks followed a general trend where the 3D‐printed and Cerrobend blocks differed between each other by approximately 1.5% on average. When compared to the TPS, both the 3D‐printed and Cerrobend blocks were found to range within 2% of the TPS. The point dose measured between the blocks followed a general trend where when measured in the open field, the 3D‐printed measurement was found to be lower than Cerrobend by approximately 0.8% on average. This can be accounted for due to the 3D‐printed photon block shell baseplate, which served as an interface to attach the tungsten BB‐filled shells to the applicator tray, with an approximate thickness of 1.6 mm PLA. This difference was found to align the 3D‐printed blocks closer to the measured TPS value. The measurement for patient B had been taken beneath the MLC leaves between the blocks, attributing to the nearly zero difference in output. The notable differences exceeding 1% between the 3D‐printed and Cerrobend blocks for both the under and between block measurements were presumed to be due to the disparity in the shape, placement, orientation, and density of the blocks. Overall, adopting institutions are able to tune their clinical TPS to reflect the 3D‐printed photon blocks over the Cerrobend standard.

The MV image profile analysis also emphasized the approximate 1% attenuation of the beam outside of the shielding regions. The notable differences exceeding 1% between the 3D‐printed and Cerrobend block were presumed to be due to the disparity in the shape, placement, and orientation of the blocks.

The photon block field geometry underscored the manual error in manufacturing and positioning of the Cerrobend photon blocks. However, the 3D‐printed photon blocks were still susceptible to potential placement error due to the manual attachment of the 3D‐printed baseplate to the tray via aluminum nuts and bolts. This error was identified in Table 4, where for patient C, the 3D‐printed photon blocks in‐field were 10.8% dissimilar to the TPS. An inspection of Figure 7 for patient C provided insight that the 3D‐printed blocks might have been attached to the plate 0.5 mm laterally, with a slight rotation. This is because the holes in the Varian supplied acrylic block tray are 7.75 mm diameter and the ¼−20 screws are 6.15 mm outer diameter in the threaded region. To eliminate this slack, future versions may utilize custom designed screws or centering rings on the screws that fill this gap, and eliminate manual alignment.

### 4.2 Advantages and limitations

The novel 3D‐printed photon block presents a range of advantages and limitations. The fully tungsten BB filled 3D‐printed block shell was found to maintain structural rigidity within 0.3 mm at all four cardinal gantry angles. While block volumes of 90 cc or less were directly investigated, the minimal sag suggests sizes of at least double are acceptable, though specific commissioning should be performed. In our commissioning examples each 3D‐printed shell also holds two of these blocks of near equal size. Another one of its major advantages lies in the maximum block fidelity for the edges and divergence. Being an entirely digital process, it allows for precision that is hard to achieve with manual processes. This eliminates the need for someone to be trained or to spend time creating photon blocks by hand, reducing manual labor associated with the manufacturing of the blocks significantly. Furthermore, it utilizes infrastructure that is already used for either 3D‐printed boluses or 3D‐printed electron cutouts, streamlining the process and making it more cost‐effective. Another notable advantage is the automation of several aspects of the process. The patient identifier, jaw orientation, field orientation (AP/PA), and block placement are all automated, reducing the chances of human error and enhancing the efficiency of the workflow. Despite these numerous benefits, the novel photon block also comes with a few limitations. There can be potential printing defects, an inherent risk with any 3D‐printing process. Careful quality control measures are needed to ensure these defects are caught and corrected. Another limitation is the 3D‐printed baseplate blocks the light field during treatment. The placement, however, is commonly verified through image guided radiation therapy prior to treatment delivery through MV images. Finally, the process requires the purchase of tungsten BBs, which is an added expense that clinics would need to consider but could also be used for 3D‐printed electron cutouts.

### 4.3 Proposed clinical workflow

After completion of thorough commissioning by a physicist, we recommend MV images of the 3D‐printed photon blocks should be taken prior to a patient's treatment and compared to the TPS for dosimetric and geometrical alignment for the first dozen patients. We then suggest the routine clinical workflow as follows: (1) The treatment planner, using the automated script, creates the STL file for 3D‐printing. (2) The physicist or physicist assistant then prints the block shell using an optimized printing profile with a 3D‐printer. (3) The physicist or physicist assistant then weighs the block shell, fills the block shell compactly with tungsten BBs, and re‐weighs the block, to obtain a tungsten BB mass within 2% of the calculated value. (4) The block is then capped, aligned properly, and attached to the acrylic tray via aluminum nuts and bolts. (5) A transparency is then printed at 55.4 cm SSD with the photon block from the TPS to verify the correct jaw orientation, shape of the printed block, patient identifier, and field orientation. (6) The block is then given to the patient's treatment machine and the acting radiation therapists. (7) After the completion of the patient's treatment, the 3D‐printed photon block and tray would be returned to the physicist or physicist assistant to be disassembled. (8) The tungsten BBs, aluminum nuts and bolts, and acrylic tray would be stored for future use, and the empty 3D‐printed photon block shell would be kept for recycling into filament, disposed of, or provided to the patient.

## 5 CONCLUSIONS

This study has validated a novel method for 3D‐printed photon block creation and use in clinical settings, showing accuracy and efficiency gains over traditional manual processes. While acknowledging potential printing defects and light field blockage, we argue that these limitations are outweighed by the benefits of reduced manual labor, automated processes, and greater precision. Following a carefully outlined clinical workflow, we envision the broader adoption of this technique in the field of radiation therapy, furthering the advancement of non‐toxic photon shielding. We hope this approach is able to be easily adopted by care teams.

## AUTHOR CONTRIBUTIONS

Joseph B. Schulz, Piotr Dubrowski, Clinton Gibson, Amy S. Yu, and Lawrie Basil Skinner contributed to the conception and design of the study. Joseph B. Schulz collected all data. Joseph B. Schulz and Piotr Dubrowski performed the data analysis. Joseph B. Schulz wrote the first draft of the manuscript. All authors contributed to manuscript revision, read, and approved the submitted version.

## CONFLICT OF INTEREST STATEMENT

Amy S. Yu and Lawrie Basil Skinner have a US patent on field shaping devices utilizing tungsten alloy ball bearings.
